# Temporal and Geographical Patterns of Pacific Arboviral Vectors on Ebeye, Republic of the Marshall Islands: Insights from a Longitudinal Entomological Study

**DOI:** 10.3390/pathogens15010060

**Published:** 2026-01-07

**Authors:** Anna A. Drexler, Tamara S. Buhagiar, Saul Lozano, Earlynta Chutaro, Calvin Juda, Roston Morelik, Janet McAllister, Limb K. Hapairai

**Affiliations:** 1 Arboviral Diseases Branch, Division of Vector-Borne Infectious Diseases, US Centers for Disease Control and Prevention, Fort Collins, CO 80521, USA; nkq3@cdc.gov (S.L.); janet.mcallister@nola.gov (J.M.); 2 Pacific Island Health Officers’ Association (PIHOA), Honolulu, HI 96960, USA; tamara.buhagiar@gmail.com (T.S.B.); rostonm@pihoa.org (R.M.); limbh@pihoa.org (L.K.H.); 3 Ministry of Health and Human Services, Majuro 96960, Marshall Islands; echutaro@rmihealth.org (E.C.); cjuda@rmihealth.org (C.J.)

**Keywords:** *Aedes aegypti*, *Aedes albopictus*, adult mosquitoes, arboviruses, eggs, mosquito abundance, ovitraps, Republic of the Marshall Islands, vector control, vector surveillance

## Abstract

Arthropod-borne viruses (arboviruses) such as dengue, chikungunya, Zika, and yellow fever pose significant global health risks, with mosquitoes from the *Aedes* genus as the primary vectors responsible for human transmission. The Republic of the Marshall Islands (RMI), particularly the urbanized areas of Kwajalein and Majuro atolls, has experienced multiple outbreaks of dengue, Zika, and chikungunya with substantial health and economic impacts. Vector control remains the most effective method for reducing disease risk, but comprehensive data on local mosquito vector composition, distribution, and abundance are needed to guide new, effective control efforts. From 2022 to 2024, we conducted a longitudinal baseline assessment of mosquito abundance and species composition on Ebeye and nearby islets in Kwajalein Atoll, RMI, using BG-Sentinel traps and ovitraps. *Aedes aegypti* was the most prevalent species, accounting for 58% of all adult females collected across study locations, with higher relative abundances on Ebeye than on northern islets (4.7 vs. 2.3 per trap/night). *Aedes albopictus* was more abundant on northern islets (0.7 vs. 3.2 per trap/night), and *Culex quinquefasciatus* showed similar abundances (1.2 vs. 1.7 per trap/night). Rainfall and anthropogenic factors, including water storage practices and housing density, influenced mosquito abundance. These findings provide multi-seasonal baseline data to support targeted vector control strategies in RMI.

## 1. Introduction

Mosquito-borne viruses (arboviruses) are responsible for disease outbreaks around the world and have long-lasting health and economic consequences. Dengue, chikungunya, Zika and yellow fever viruses, which are all transmitted by *Aedes* species, put at risk more than 3.9 billion people each year, resulting in 100–400 million infections and 100 million cases annually [1]. Changes in ecological conditions have altered the distribution of vectors, changed their life cycles and activities, and disrupted host-pathogen dynamics, causing more frequent and severe arboviral outbreaks [2,3,4]. Factors such as global migration, urbanization, and population displacement impact the spread of arboviral diseases, increasing their incidence and geographical reach [5].

The Pacific region has experienced multiple *Aedes*-borne arboviral disease outbreaks, with 104 incidents reported in 19 of the 22 Pacific Island countries (PICs) from 2014–2020, including 72 dengue, 14 chikungunya, and 18 Zika outbreaks [6]. Since 2015, RMI has had significant outbreaks of chikungunya (1317 cases), Zika (34 cases), and dengue (3446 cases). Among its 29 atolls, outbreaks historically started in the most populated–Kwajalein (9789 residents) and Majuro (23,156 residents)–which together constitute 78% of the RMI’s total population. Ebeye Island, with an estimated 41,667 people per square kilometer, is the most densely populated island in Kwajalein Atoll, in the Pacific, and the sixth in the world [7,8]. The most recent dengue outbreak in the RMI (August 2019–June 2021) originated in Kwajalein Atoll and later moved to Majuro before it reached outer island areas [9]. Of the 3884 dengue-like illness cases reported (DENV-3, 2019–2021), 277 and 3401 occurred in Ebeye and Majuro, respectively. These areas were most affected due to their high population densities and urban settings, which favor the proliferation of *Aedes (A.) aegypti* (Linnaeus, 1762) and *Aedes albopictus* (Skuse, 1894), invasive mosquitoes of significant epidemiologic concern [10].

Adult *Aedes* spp. females can acquire an arbovirus when feeding from an infectious host [11]. Female mosquitoes blood-feed multiple times from different hosts, increasing their likelihood of acquiring and transmitting a circulating arbovirus [12,13]. Gravid females then deposit eggs in containers in or near human habitation, which develop through larval and pupal stages, emerging as adults [14,15]. *Aedes albopictus* exhibits opportunistic feeding behavior with a preference for humans and lays eggs in habitats ranging from sylvatic to peri-urban and urban [16]. *Aedes aegypti* feeds primarily on humans and lays eggs in artificial containers in and around urban dwellings [17]. *A. aegypti* and *albopictus* typically have 3–5 gonotrophic cycles during their lifespans, which average 25–30 days under tropical conditions [18,19,20,21,22]. Mosquitoes cycle through host-seeking, blood-feeding, and oviposition stages throughout this period.

Understanding local mosquito populations is important for developing effective control strategies to reduce the risk of arboviral outbreaks. Entomological surveys can provide valuable insights into species composition and abundance but are typically short in duration and reflect a specific point in time. Surveys from the 1940s in Kwajalein Atoll revealed four mosquito species: *A. aegypti*, *A. marshallensis* Stone and Bohart, *Culex (C.) quinquefasciatus* Say, and *C. annulirostris* Skuse [23]. More recent surveys of nonurbanized Kwajalein islets found *A. albopictus* and mosquitoes from the *A. scutelaris* complex but not *A. aegypti*, *A. marshallensis*, or *C. quinquefasciatus* [24]. In Majuro Atoll, home to RMI’s capital Majuro and urban center Delap-Uliga-Djarrit, both adult *A. aegypti* and *A. albopictus* were found across all the sampled locations, with immature *A. aegypti* in 94% of the domestic containers surveyed [25]. Despite its high population density and role in recent arboviral outbreaks, there are no similar published studies from Ebeye detailing mosquito species composition, distribution, and abundance over time.

This study aims to fill this knowledge gap by conducting longitudinal baseline mosquito surveillance on Ebeye to characterize arboviral vector populations and examine how human and environmental factors influence abundance and species composition over time. These findings provide insights into the dynamics of vector populations, which are essential for informing both traditional and innovative mosquito control strategies to reduce vectors and risk for outbreaks of human arboviral diseases.

## 2. Materials and Methods

### 2.1. Study Site and Population

**Figure 1:**
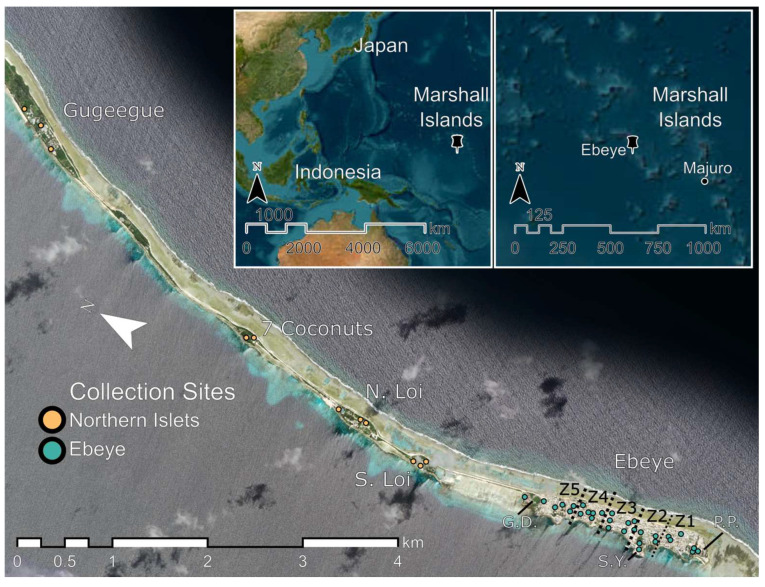
Map of Ebeye and northern islets, Kwajalein Atoll, Republic of Marshall Islands with surveillance sites noted; G.D. denotes garbage dump; S.Y. denotes shipping yard; P.P. denotes power plant; municipal zones are delimited with dashed lines and denoted as Z1, Z2, Z3, Z4, and Z5.

The RMI occupies 181 km^2^ of Micronesia and comprises more than 1200 islands and islets in two chains of coral atolls, Ralik and Ratak. Kwajalein Atoll (8°43′ N, 167°43′ E), which is part of the Ralik chain, is one of 29 atolls in the RMI and comprises 97 islets surrounding one of the largest lagoons in the world. Ebeye is the most populated island on Kwajalein Atoll and the second largest urban population center in the RMI. The current study focused on Ebeye and four adjacent “northern” islets connected via a land bridge: Gugeegue, Seven Coconuts, North Loi, and South Loi (Figure 1).

The average elevation is 2 m above sea level. The climate is tropical with >2500 mm of rainfall per year, primarily from May–November. Ebeye has an area of 0.36 km^2^ and is predominantly urban, with a population of 9789 and approximately 1300 households [7]. Daily average temperature was 27 °C with a standard deviation (sd) of 0.55 °C; averaged daily minimum temperature was 27 °C with sd of 0.62 °C. Ebeye has minimal vegetation, while the northern islets are sparsely populated with plenty of natural vegetation, serving as a point of comparison for Ebeye. In 2019, entomological surveillance during the RMI’s dengue outbreak response (McAllister and Rose, CDC, unpublished) revealed the presence of *A. aegypti* and *A. albopictus* on Ebeye with mosquito larval habitats, including artificial containers and natural habitats (leaf axles, coconut shells, etc.). A desalination plant supplies fresh water, but rainwater remains a primary resource on Ebeye, and 79% of households rely on collected rainwater stored in water catchments for household needs. These storage practices provide suitable conditions for mosquito breeding.

### 2.2. Study Design

From May 2022–September 2024, we collected adult mosquito data from Ebeye and four adjacent northern islets (Gugeegue, Seven Coconuts, North Loi, and South Loi). From May–December 2022, data were collected from 25 sites: 14 on Ebeye and 11 on the northern islets. The target trapping density of ~1 trap/ha was reached by the week of 12 December 2022, with a minimum distance of 50–100 m between trapping locations. This density is commonly used in vector surveillance programs and was selected to balance adequate spatial coverage with operational feasibility for a long-term study. From December 2022 through September 2024, 41 collection sites were used, with 30 evenly distributed across Ebeye’s five municipal zones (six per zone) and 11 on the northern islets–Gugeegue (3), North Loi (3), and South Loi (3), and Seven Coconuts (2). Key surveillance sites on Ebeye included the power plant, shipping yard, and garbage dump, which are located in Zones 1, 2, and 5, respectively (Figure 1), and were prioritized to ensure highly *Aedes aegypti* productive habitats were sufficiently surveyed over the course of the study.

### 2.3. Mosquito/Egg Sampling and Processing

A laboratory space in Ebeye Hospital was allocated by the Ebeye-based Ministry of Health and Human Services (MOHHS) for initial project use. As part of the project, a mosquito laboratory was commissioned on the Ebeye Hospital grounds, providing office, wet-lab and storage areas, and a fully equipped insectary.

Adult mosquitoes were collected weekly over a 24 h sampling period via BioGents-Sentinel II traps baited with BioGents-Lure (BGS and BG-Lure, Biogents AG, Regensburg, Germany) and connected to a 12-volt battery. BGS traps were placed outside homes in peri-domestic areas, where possible under the cover of vegetation or awnings. A minimum distance of 50 m was maintained between traps. BGS trap collection bags containing mosquitoes were returned from the field to the laboratory and stored in a freezer at −18 °C. Mosquitoes were sorted from bycatch before identification. For each trap collection, mosquitoes were sexed, counted, and identified to species (*A. aegypti*, *A. albopictus*, *A. marshallensis*, *C. annulirostris*, and *C. quinquefasciatus*) via WRBU species keys [26]. Mosquitoes not identifiable to species were recorded by genus (*Aedes* or *Culex*) or as unidentifiable.

From May 2022–August 2023, ovitraps were set each week to collect *Aedes* spp. eggs. Ovitraps were 500 mL black plastic cups (16oz/17oz Stadium Cups, Vistaprint, Venlo, Netherlands) containing a 15 cm × 2.5 cm textured particle board paddle (Hardboard, Eucatex, Alpharetta, GA, USA) filled with ~300 mL tap water. The ovitraps were placed near BGS trap locations but were set on days when BGS traps were not deployed. After 5 days of collection, ovitraps were emptied of water, and paddles retrieved from the field for counting the deposited *Aedes* mosquito eggs. Eggs were not hatched for species identification and were frozen at −18 °C for 48 h. before being discarded.

### 2.4. Meteorologic Data

The temperature, wind and rainfall data were obtained from the ERA5 post-processed daily statistics weather data product [27].

### 2.5. Data Analysis

We used a negative binomial distribution (NB) to estimate the relative abundance of adult females captured for each mosquito species in Ebeye and northern islets. Relative abundance was defined as the number of mosquitoes of each species collected per trap per night [28]. We parameterized the NB on the mean, following standard ecological practices, without any covariates with data from May 2022 through September 2024).

We used an additional general linear mixed model (GLMM), with data from the 41 traps (end of December 2022 through September 2024), to understand how two known environmental covariates, rain and urbanization, in addition to location and time (with site and week included as random effects), influence mosquito catches. Temperature was not included because its variation was very low. The mean of the daily average temperature was 27 °C with a standard deviation of 0.55 °C. Also, the daily minimum temperatures were well above the activity threshold for the species [29]; averaged daily minimum temperature was 27 °C with a standard deviation of 0.62 °C. The model also partitioned total variability in mosquito catches (*C*) into spatial ($\sigma_{site}^{2}$), temporal ($\sigma_{week}^{2}$) and spatiotemporal components ($\sigma^{2}$), as follows:

$$C_{i,t}\sim Poisson(\lambda_{i,t})$$

$$log\left(\lambda_{i,t}\right)=\mu +\beta_{1}\cdot \log\left({Rain}_{t}\right)+\beta_{2}\cdot {Wind}_{t}+\beta_{3}\cdot {Ebeye}_{i}+{site}_{i}+{week}_{t}+\epsilon_{i,t}$$

$${site}_{i}\sim Normal(0,\sigma_{site}^{2})$$

$${week}_{t}\sim Normal(0,\sigma_{week}^{2})$$

$$\epsilon_{i,t}\sim Normal(0,\sigma^{2})$$

We modeled adult female mosquito catches at each *site* (*i*) and *week* (*t*) via a Poisson distribution with a mean relative abundance ($\lambda_{i,t}$), accounting for extra variation in the *site*, *week* and *ε* parameters. In this model, *µ* was a grand mean and the baseline abundance when the other parameters were zero. We used a 14-day (right) rolling average of rainfall, log-transformed for better fit, and average wind speed during collection as key factors. A 14-day period is commonly applied in entomological models, and reflects the time required for *Aedes* mosquitoes to develop from egg to adult (~7 days) plus the time from blood meal through oviposition (~7 days). Ebeye is more densely populated than the northern islets; therefore, a binary covariate was used to distinguish between traps in each location (Ebeye = 1 and Ebeye = 0, respectively). The models included fixed effects for rainfall, wind, and whether the collection took place in Ebeye, as well as random effects to account for variability across sites, weeks, and additional sources of variation.

We opted to use Bayesian inference to fit the selected models to the data, to follow the American Statistical Association’s recommendation to avoid using *p*-values as dichotomous decision tools, and to adopt richer approaches to uncertainty (Wasserstein and Lazar, 2016). Fitting was conducted in R (version 4.4.3) [30] with the help of the package jagsUI (version 1.6.2) [31], a library that interacts with JAGS (version 4.3.2) [32]. The fit of the models was evaluated using the coda library (version 0.19-4.1) [33] and recommended best practices. Model fit was assessed by $\hat{R}$ < 1.01, with Effective Sample Sizes > 100,000, and visually with trace plots generated by the coda package. Further statistical details can be found in the Supplementary Materials.

### 2.6. Ethical Considerations

The overall study protocol was reviewed and approved by the RMI MOHHS Institutional Review Board Committee. Consenting residents and business owners signed a form under the RMI MOHHS granting permission for the field team to enter the property to set and collect BGS and ovitraps weekly throughout the study. Participation was voluntary, with no compensation provided. A total of 41 households were recruited. The study design included both adult and egg mosquito collection via noninvasive trapping methods that cause no harm to the participants or the environment. Data analysis involved statistical modeling techniques to explore mosquito abundance patterns. The privacy and confidentiality of individual participants were maintained throughout the analysis process.

## 3. Results

### 3.1. Abundance and Species Composition Across the Study Area

**Figure 2:**
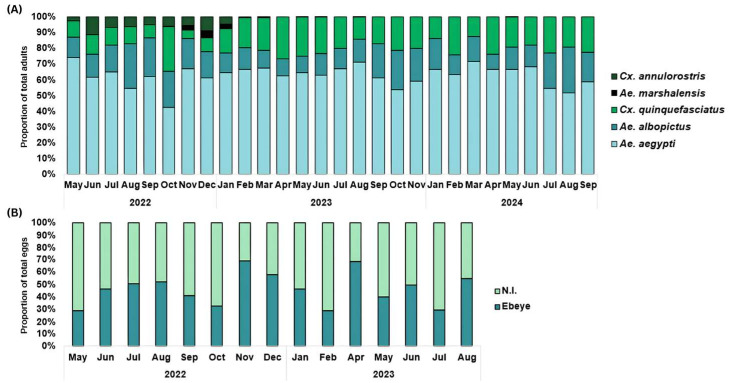
Adult and egg trapping. (**A**) Relative proportions of mosquito species collected in BGS traps from May 2022–September 2024. (**B**) Relative proportions of eggs collected from the Ebeye and northern islet (N.I.) locations from May 2022–August 2023.

From May 2022 through September 2024, 3883 BGS collections were made: 2780 from 30 trap sites on Ebeye and 1103 from 11 trap sites on the northern islets, with 81% (n = 3145) of collections having at least one female mosquito. A total of 25,804 female mosquitoes were captured, 17,841 (69%) from Ebeye and 7963 (31%) from the northern islets. Table 1 shows the relative abundance of all species collected from Ebeye and northern islets via BGS trapping. Five species were identified in the BGS traps, with *A. aegypti* being the most prevalent (58%), followed by *A. albopictus* (20%), *C. quinquefasciatus* (19%), *C. annulirostris* (2%), and *A. marshallensis* (0.5%), and their relative proportions in the BGS traps over time are shown in Figure 2. Additional mosquito species were not detected in the BGS samples. We observed significant differences in the mean number of females per trap per night between Ebeye and northern islets for *A. aegypti*, *A. albopictus*, *A. marshallensis*, *C. quinquefasciatus*, which ranged from 2- to 8-fold, whereas *C. annulirostris* remained similar across the study areas.

Ovitrap samples were collected weekly from 41 sites (30 on Ebeye, 11 across 4 northern islets) from December 2022–August 2023, and more than half of the 1243 ovitraps sampled were positive for one mosquito egg or more. Sixty-one percent (n = 756) of the samples were collected from Ebeye, 36% (n = 445) from northern islets, and 49% and 71% were positive for eggs, respectively. Over the course of the study, 13,708 total eggs were collected, roughly half (48%, n = 6540) from Ebeye and from the northern islets (53%, n = 7257). The number of eggs per trap per night was almost 2-fold greater on the northern islets than on the Ebeye islets, with mean egg abundance values of 17.2 [CrI 14.5, 19.9] versus 9.1 [CrI:7.5, 10.6], respectively.

**Figure 3:**
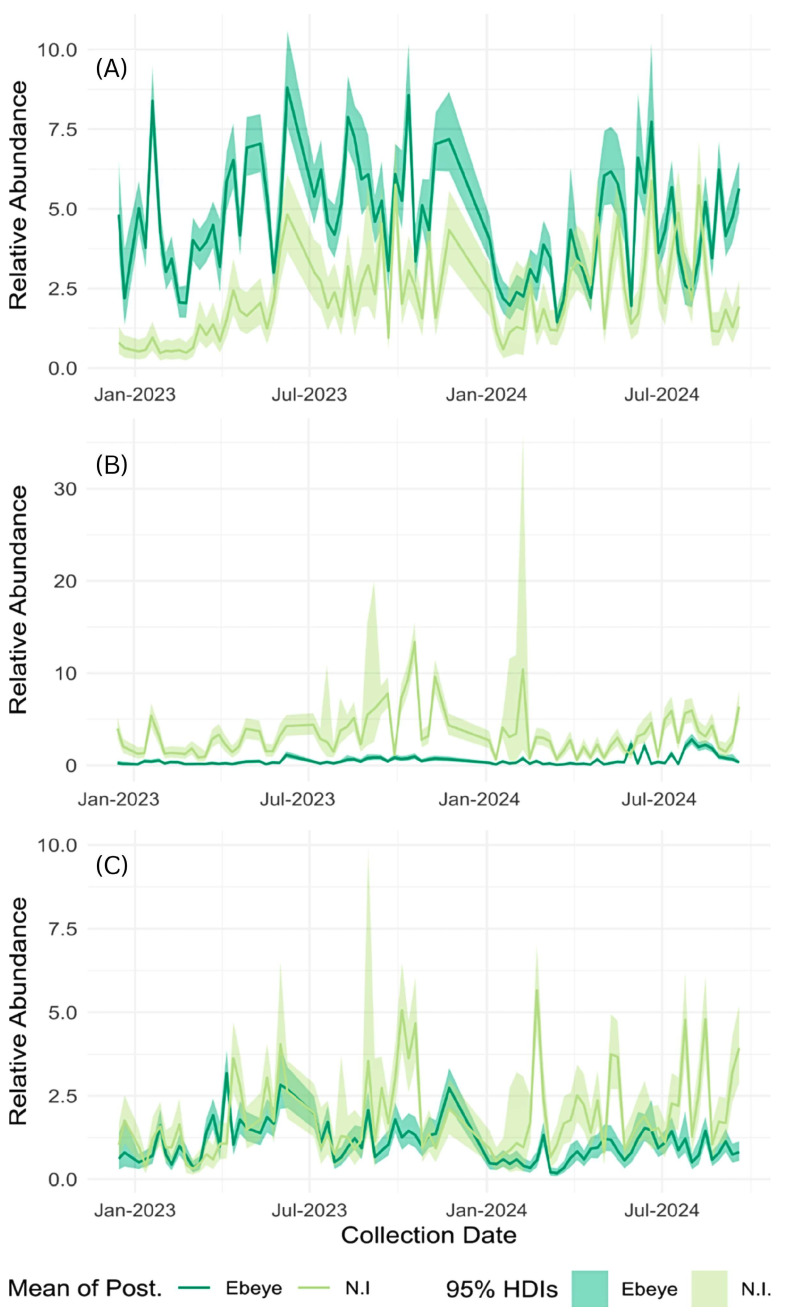
Estimated relative abundances of adult female *A. aegypti* (**A**), *A. albopictus* (**B**) and *C. quinquefasciatus* (**C**) from January 2023–September 2024.

Figure 3 shows the relative abundance of female *A. aegypti*, *A. albopictus*, and *C. quinquefasciatus* collected across the study areas. Ebeye had consistently greater absolute numbers of adult female *A. aegypti* than did the northern islets. Among all the *A. aegypti* collected, 84% (n = 12,588) were found on Ebeye, and only 16% (2491) on the northern islets. Their estimated relative abundance was also significantly greater in Ebeye, at 4.7 per trap per night [CrI:4.4, 4.9], than in northern islets, at 2.3 [CrI:2.0, 2.5]. The peak trap abundances of female *A. aegypti* on Ebeye were more than double those found on the northern islets (96 vs. 43 females). Conversely, fewer *A. albopictus* were collected from Ebeye (n = 1817, 34%) than from northern islets (n = 3439, 65%), and the estimated mean abundances were also lower—0.7 [CrI:0.5, 0.7] verses 3.2 [CrI:3.0, 3.8]. At their peak, the trap abundances for female *A. albopictus* on Ebeye were less than half (0.4-fold) those on northern islets (27 vs. 66 females).

Among all the adult female *C. quinquefasciatus* collected, 62% (n = 3101) were from Ebeye, and 37% (n = 1845) were from northern islets, and the estimated mean relative abundance was 1.2 [CrI:1.1, 1.2] for Ebeye and 1.7 [CrI:1.5, 1.9] for the northern islets, indicating higher abundances in the latter area. Despite this, instances of high numbers of female *C. quinquefasciatus* in BGS traps occurred on Ebeye, with peak trap abundances 4-fold greater than those found in northern islet collections (236 vs. 60 per trap per night). Although *C. annulirostris* (n = 427) was initially identified in the BGS traps, this species was not found in the samples collected after April 2023. *A. marshallensis* was collected in low numbers over the course of the study (n = 120) and predominantly on the northern islets, despite fewer overall collections in this area. The estimated mean abundance for this species was 0.01 [CrI:0.003, 0.02] on Ebeye verses 0.08 [CrI:0.03, 0.13] on northern islets.

### 3.2. Environmental Drivers of Mosquito Abundance

**Figure 4:**
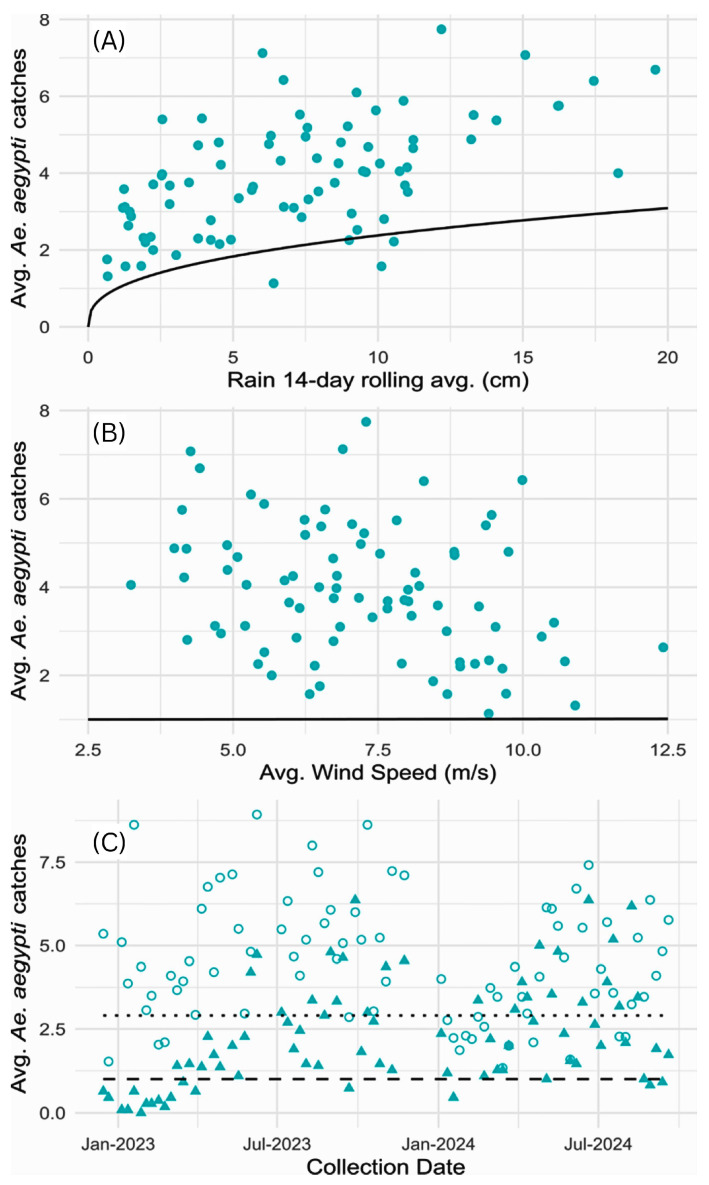
Effects of weather variables on the abundance of female *A. aegypti*. (**A**) Rain: the black line represents the adjusted and linearized *β*_1_ log(Rain) parameter. (**B**) Wind: The black line represents the *β*_2_ × Wind parameter. (**C**) Location: Open circles represent collections made in Ebeye and filled triangles collections from the northern islets over the 2022–2024 collection period. The dotted line shows the predicted values for Ebeye (*β*_3_ × Ebeye = 1), while the dashed line shows the predicted values for the northern islets (*β*_3_ × Ebeye = 0).

Generalized linear mixed models (GLMMs) were used to assess the effects of rainfall, wind, and site-specific factors on the abundance of females of all species (Table 2 and Figure 3 and Figure 4). Rainfall significantly increased abundance, with estimated effect sizes of 1.3 [CrI:1.2, 1.4], 1.3 [CrI:1.1, 1.6] and 1.4 [CrI:1.2, 1.5] for *A. aegypti*, *A. albopictus* and *C. quinquefasciatus*, respectively. This corresponds to an approximate 30–35% increase in abundance for each species when rainfall doubles (e.g., from 5 to 10 cm). Wind had no significant effect on mosquito abundance for any species. For *Aedes marshallensis* and *C. annulirostris*, Markov chain Monte Carlo simulations failed to converge, and the models do not reliably describe the data; thus, results for these species are not presented.

Female *A. aegypti* abundance was roughly 200% greater for Ebeye, with an estimated effect size of 3.04 [CrI:1.6, 4.6]. In contrast, *A. albopictus* abundance was ~91% lower for Ebeye, with an estimated effect size of 0.09 [CrI:0.04, 0.14]. Site- and week-level variability estimates were significant for both *Aedes* species: *A. aegypti* (σ^2^_site = 2.0; σ^2^_week = 1.4) and *A. albopictus* (σ^2^_site = 2.5; σ^2^_week = 2.0), indicating their populations fluctuated substantially over space and time even after accounting for rain, wind and location. *C. quinquefasciatus* abundance was lower for Ebeye (0.5, CrI:0.2, 0.9), and also showed high spatial and temporal variability ($\sigma_{site}^{2}$ = 2.3; $\sigma_{week}^{2}$ = 1.6). Notably, residual variance was higher in the *Culex* models than for *Aedes aegypti* (Supplementary Files), indicating significant population effects unexplained by spatial, temporal or environmental variables.

## 4. Discussion

This study provides the first detailed baseline assessment of mosquito abundance and species composition on Ebeye and its connected northern islets on Kwajalein Atoll, RMI. *Aedes aegypti* was the predominant species (58% of all female mosquitoes) and the main vector on Ebeye, likely due to the high degree of urbanization, availability suitable container habitats and dense human population. *Aedes albopictus* (20%) was mainly found in rural and suburban areas of the northern islets, explained by its preference for vegetated environments. *Culex. quinquefasciatus* (19%) was distributed across urban and rural areas, consistent with other reports in RMI and Pacific region [23,25,34,35].

The current study is the first published report of *A. aegypti* on Ebeye, confirming unpublished surveys after Ebeye’s 2018–2019 dengue outbreak that found *A. aegypti* associated with urbanized areas and domestic breeding containers (McAllister and Rose, CDC, unpublished). Prior to this, only a 1972 survey reported *A. aegypti* from Kwajalein Atoll, on Kwajalein Island—home to a US military base and airport. Subsequent surveys did not detect this species [34], potentially due to housing type and vector management activities on the base. High prevalence of *A. aegypti* has also been detected in other urban areas of RMI, notably in Majuro associated with 2011 dengue [35] and 2016 Zika outbreaks [25]. Since *A. aegypti* is a primary vector of arboviruses, its establishment on Ebeye represents a significant risk for transmission in this area.

*Aedes albopictus* abundance and distribution patterns from this study are consistent with reports from Kwajalein Atoll [24,34], Majuro [25], and central Pacific [23] that show this species prefers peri-urban/rural vegetated environments and utilizes both natural (e.g., tree holes, leaf axils) and artificial container larval habitats, explaining its low abundance in Ebeye. Similar to studies from RMI and the broader Pacific, *C. quinquefasciatus* was widespread in human-associated and natural habitats, contributing to persistent mosquito populations [23]. *Culex quinquefasciatus* habitats in Marshall Islands include water drums and catchment systems (McAllister and Rose, CDC, unpublished), artificial containers [35] and discarded tire piles [25,34]. *Aedes marshallensis*, native to RMI and found in Federated States of Micronesia and Kiribati [36,37], was also present in low numbers, but its arbovirus competence is not well understood and its potential role in outbreaks requires further investigation [37].

Despite similar total adult *Aedes* abundances across study locations, ovitrap positivity rates and eggs per trap/night were higher on the northern islets compared with Ebeye. This could be explained by differences in oviposition site availability and species-specific preferences. Based on our observed adult species compositions, eggs collected from Ebeye were likely primarily *A. aegypti*, while the eggs from northern islets were likely mainly deposited by *A. albopictus*, with some contributions from *A. aegypti* and *A. marshallensis*. Ebeye has abundant container habitats in and near homes that could provide alternative, competing oviposition sites from the ovitraps (McAllister and Rose, CDC, unpublished), leading females to distribute eggs across multiple sites and resulting in fewer eggs per ovitrap. Container-site preferences may also contribute: studies suggest *A. aegypti* prefer larger containers [38,39], consistent with local observations from Ebeye (McAllister and Rose, CDC, unpublished) and Majuro [25] where barrels/drums and cisterns were productive *A. aegypti* habitats. In contrast, *A. albopictus* may prefer smaller containers in highly vegetated areas and shows similar attraction to ovicups and natural habitats [40,41,42]. Finally, interspecific interactions could have influenced the attractiveness of oviposition sites [43,44], potentially contributing to the high abundances and ovitrap positivity seen in northern islets. Together, these factors underscore the complex ecological processes that shape oviposition behavior in mixed-species environments.

In the current study, rainfall was a significant driver of mosquito abundance across species, consistent with previous studies showing the influence of weather on mosquito dynamics and increased arboviral risk in Pacific and other small island contexts [45,46,47]. For example, in Puerto Rico, rainfall was linked with increased *A. aegypti* densities, heightened egg-laying activity in discarded tires and water storage containers, and spikes in dengue incidence [48]. Similarly, models for Hawaii predict that above-average precipitation will expand the natural habitat of *A. albopictus* across Pacific islands, increasing the potential for arboviral outbreaks [47]. However, climatic variables alone cannot account for arboviral disease dynamics [46]. Human behaviors are important in modulating the relationship between rainfall and vector density, with peridomestic water storage in particular helping sustain larval habitats during drier seasons and resulting in more stable mosquito populations [48,49,50]. In Ebeye, there is widespread reliance on water storage and catchment systems, as well as numerous household containers which could serve as productive habitats year-round, potentially accounting for the relatively lower variance observed for *A. aegypti* in this study compared with *A. albopictus*. Given its extreme population density, Ebeye is particularly susceptible to arboviral outbreaks, highlighting the need for sustainable vector control strategies tailored to the unique challenges of urban and rural settings in the RMI [8,51,52].

This study has several limitations that might affect the interpretability of the findings. The geographic scope was limited to Ebeye and nearby northern islets, restricting generalizability to other islands and atolls in the RMI, which might differ ecologically and socioeconomically, potentially leading to distinct variations in mosquito populations and ecological dynamics. While the survey provides two years of baseline entomological data from May 2022–September 2024, the target trapping density of 1 trap/ha was met only at the end of December 2022, potentially underestimating mosquito abundances early in the study. This was addressed in data analysis via use of uncertainty metrics: the GLMM for evaluating environmental covariables used data with one trap per ha. Further, we note temporal limitations with this study—a longer surveillance timeframe (>5 years) would allow for deeper insights into longer-term ecological shifts in mosquito populations rand would likely reduce uncertainty intervals and the substantial spatial and temporal variance observed. As field entomology capacity in Ebeye improved over time, data accuracy early in the study may have been less reliable. For example, *C. annulirostris* was detected in high numbers during the first six months of sampling but not at all later in the study, raising questions about the reliability of early sampling and identification for this species, which has been previously reported in this region of the Pacific and RMI [25]. Additionally, the inability to hatch eggs precluded definitive ovitrap species identification, complicating interpretations in habitats with multiple species. Future studies to accurately identify species from ovitraps would shed light on *Aedes* spp. population dynamics in RMI. Despite these challenges, this study offers insights into mosquito ecology and supports vector control efforts in the region.

## 5. Conclusions

The collection of detailed, multiyear entomological data is important for understanding mosquito population dynamics and designing effective vector control strategies. The baseline entomological data gathered in this study provide insight into local spatial and temporal dynamics of key mosquito species—*A. aegypti*, *A. albopictus*, and *C. quinquefasciatus*—in Ebeye and connected northern islets on Kwajalein Atoll. We show that *A. aegypti* is abundant in all populated areas of Ebeye, creating ideal conditions for an introduced arbovirus to spread. Environmental management and improvement of housing and water infrastructure could reduce mosquito breeding sites, preventing sustained cross-seasonal vector populations. These findings lay a foundation for evaluating future sustainable, targeted strategies such as sterile or genetic mosquito control methods to further reduce populations and mitigate arbovirus transmission and outbreaks of vector-borne disease.

## Figures and Tables

**Table 1:** Descriptive statistics of adult mosquito and egg collections via BGS and ovitraps from two distinct areas of Kwajalein Atoll, RMI, May 2022–September 2024.

	All Sites		Ebeye			Northern Islets		
	n	%	n	% ^2^	**Mean** ^3^ [CrI]; *dispersion*	n	% ^2^	**Mean** ^3^ [CrI]; *dispersion*
**BGS collections, all**	**3883**		**2780**			**1103**		
BGS collections > 0 (+)	3145	81	1975	71		949	86	
**Total number females ^1^**	**25,804**	**100**	**17,841**	**100**		**7963**	**100**	
*A. aegypti* *	15,079	58	12,588	71	**4.7** [4.4, 4.9]; *0.64*	2491	31	**2.3** [2.0, 2.5]; *0.33*
*A. albopictus* *	5256	20	1817	10	**0.7** [0.6, 0.7]; *0.12*	3439	43	**3.2** [2.9, 3.4]; *0.46*
*A. marshallensis* *	120	0.5	35	0	**0.01** [0.003, 0.02]; *0.003*	85	1	**0.1** [0.03, 0.1]; *0.01*
*C. annulirostris*	427	2	305	2	**0.2** [0.1, 0.3]; *0.03*	112	1	**0.2** [0.1, 0.3]; *0.05*
*C. quinquefasciatus*	4946	19	3101	17	**1.2** [1.1, 1.2]; *0.28*	1845	23	**1.7** [1.5, 1.9]; *0.24*
Unidentified	18	>0.01	7	>0.01	NA	11	>0.01	NA
**Ovitrap collections, all**	**1243**		**756**	**100**		**445**	**100**	
Ovitrap collections > 0	684	55	370	49		316	71	
Total number eggs	13,708		6540	48	**9.1** [7.5, 10.6] ^4^; *0.19*	7257	53	**17.2** [14.5, 19.9] ^4^; *0.39*

^1^ Refers to the number of females in the BGS traps; ^2^ Percentage comparisons within location group (e.g., total, Ebeye, northern islets); ^3^ Estimated mean relative abundance, 95% credibility intervals [CrIs]; ^4^ Eggs per trap per “work week”; * Indicates a >95% significant difference between the Ebeye and northern islets; NA: Not applicable; relative abundance not estimated for unidentified samples.

**Table 2:** GLMM results for female vector abundance by species (*Aedes aegypti*, *Aedes albopictus*, and *Culex quinquefasciatus*) across urban and remote areas of Kwajalein Atoll, Marshall Islands.

Species/Parameter	Estimate (Log Scale)	B. Trans. Estimate ^1^	95% CrI of B. Trans. Estimate	Interpretation
*Aedes aegypti*				
Intercept	−0.9	0.4	0.2–0.8	When all covariates were zero abundance was 0.4
*β*_1_ (log(Rain)) *	0.4	1.3	1.2–1.4	Doubling of rain increases the abundance by ≈30%
*β*_2_ (Wind)	0.0	1.0	0.95–1.1	No effect
*β*_3_ (Ebeye)	1.1	3.0	1.6–4.6	Ebeye increased the abundance by ≈200%
Variance Components				
$\sigma_{site}^{2}$	0.7	2.0	1.7–2.4	Positive effect
$\sigma_{week}^{2}$	0.3	1.4	1.3–1.5	Positive effect
$\sigma^{2}$	0.7	2.0	1.7–2.4	Positive effect
*Aedes albopictus*				
Intercept	0.02	1.2	0.2–2.9	
*β*_1_ (log(Rain)) *	0.4	1.3	1.1–1.6	Doubling increases the abundance by ≈33%
*β*_2_ (Wind)	−0.1	0.9	0.8–1.0	
*β*_3_ (Ebeye)	−2.5	0.09	0.04–0.14	Ebeye decreased the abundance by ≈91%%
Variance Components				
$\sigma_{site}^{2}$	0.9	2.5	2.0–3.1	Positive effect
$\sigma_{week}^{2}$	0.7	2.0	1.8–2.3	Positive effect
$\sigma^{2}$	0.9	2.5	2.0–3.1	Positive effect
*Culex quinquefasciatus*				
Intercept	−1.3	0.3	0.1–0.6	
*β*_1_ (log(Rain)) *	0.5	1.4	1.2–1.5	Doubling increases the abundance by ≈35%
*β*_2_ (Wind)	0	1.0	0.9–1.1	No effect
*β*_3_ (Ebeye)	−0.7	0.5	0.2–0.9	Ebeye decreased the abundance by ≈50%
Variance Components				
$\sigma_{site}^{2}$	0.8	2.3	1.9–2.8	Positive effect
$\sigma_{week}^{2}$	0.4	1.6	1.4–1.7	Positive effect
$\sigma^{2}$	0.8	2.3	1.9–2.8	Positive effect

^1^ Backtransformed; * Rain was input as log(Rain). For interpretability, we report the backtransformed estimate 2^*β*_1_, representing the multiplicative change in expected abundance when rain doubles.

## Data Availability

All relevant data are within the manuscript and its Supporting Information Files.

## Supplementary Materials

The following supporting information can be downloaded at: https://www.mdpi.com/article/10.3390/pathogens15010060/s1. We provide the raw data, describe the algebra, and show how to recreate some of the results in the article. Table S1: *Ae. aegypti* Regression Table. Table S2: *Ae. albopictus* regression Table. Table S3: *Cx. quinquefasciatus* regression Table. Figure S1: Abundance dynamics in Ebeye and the North Islets. Figure S2: Modeled rain, wind, and location vs *Ae. aegypti* observed catches.

## Abbreviations

The following abbreviations are used in this manuscript:

A.	Aedes (used for species such as *A. aegypti*, *A. albopictus*, *A. marshallensis*)
Ae.	Aedes
BGS	BG-Sentinel trap(s)
CDC	U.S. Centers for Disease Control and Prevention
C.	Culex (used for species such as *C. quinquefasciatus*, *C. annulirostris*)
MOHHS	Ministry of Health and Human Services, Republic of the Marshall Islands
RMI	Republic of the Marshall Islands
