# Supplementary material for: Temporal and Geographical Patterns of Pacific Arboviral Vectors on Ebeye, Republic of the Marshall Islands: Insights from a Longitudinal Entomological Study
Source: Pathogens. 2026 Jan 7;15(1):60. doi: 10.3390/pathogens15010060 (PMC12845111; doi:10.3390/pathogens15010060)
Supplement: Supplementary file 1 [file pathogens-15-00060-s001.zip › pathogens-4028840-supplementary.pdf]

Supp: Temporal and geographical patterns of Pacific arboviral vectors on Ebeye, Republic of the Marshall Islands: insights from a longitudinal entomological study

Buhagiar and Drexler, et al

Introduction

We provide the raw data, describe the algebra, and show how to recreate some of the results in the article using this supplemental file. The source data is within the corresponding jagsUI model object and can be retrieve as follow:

```
library(jagsUI)

species_data <- species_model$data
```

The model description as evaluated by jagsUI can be retrieve as follow:

```
library(jagsUI)

my_model <- species_model$model
```

As mentioned in the article, to estimate the relative abundance of the species found in Ebeye (EB) and Northern Islets (NI), Kwajalein Atoll, we selected a Generalized Linear Mixed Model (GLMM).

GLMM to Partition Variability in Relative Abundance

The model allows to partition the total variability observed in the mosquito catches into a spatial, a temporal, and a spatio-temporal component. Algebraically is described as:

$$
\begin{aligned}
\mathrm{C}_{i,t} &\sim \mathrm{Poisson}(\lambda_{i,t}) \\
\log(\lambda_{i,t}) &= \mu + \beta_1 * \log(\mathrm{Rain}_t) + \beta_2 * \mathrm{Wind}_t + \beta_3 * \mathrm{Ebeye}_i + \\
&\quad site_i + week_t + \epsilon_{i,t} \\
site_i &\sim \mathrm{Normal}(0, \sigma^2_{site}) \\
week_t &\sim \mathrm{Normal}(0, \sigma^2_{week}) \\
\epsilon_{i,t} &\sim \mathrm{Normal}(0, \sigma^2)
\end{aligned}
$$

The mosquito catches ($\mathrm{C}_{i,t}$) were assumed to followed a Poisson distribution with relative abundance $\lambda_{i,t}$ at site i and week t. μ was the grand mean (intercept), Rain_t was a 14-day rolling average of the precipitation to the collection day (log-transformed), Wind_t was the average wind speed on the day of collection. Ebeye island had a larger human density than the North Islets, accordingly, when the trap was located in Ebeye the binary covariate Ebeye = 1 and when located in the NI Ebeye = 0.

The covariate $site_i$ was the random spatial effect assumed to be drawn from a zero-mean Normal distribution with variance σ^2_{site}, $week_t$ was the random temporal effect also assumed to be drawn from a zero-mean Normal distribution with variance σ^2_{week}, and $\epsilon_{i,t}$ was the residual that accommodates additional variability (overdispersion) at the spatio-temporal level. With this approach we assessed how much of the total variability was explained by space, time, and additional dispersion.

Finally, the mean relative abundance in week t for both areas was estimated as:

$$\mathrm{Abundance}_t = \mathrm{Mean}(\lambda_{i,t}).$$

Ebeye's mean abundance was estimated selecting collections with an index, i.e. $i = 12 : 41$. For the NI the index was changed accordingly.

MCMC set up

All estimated parameters used uninformative priors. The readers can expect small variations in the results they generate with this script when compare to the article.

Results recreation

The models for *Ae. aegypti*, *Ae. albopictus* and *Cx. quinquefasciatus* converged for all parameters and had an effective size of at least 10,000. However, *Aedes marshallensis* and *Culex anulorostris* did not converge, likely the result of small sample sizes.

Abundance

In Figure S1 we present the relative abundance for the three species with a converging model. For a description of this figure see article.

```
Warning: Removed 1 row containing missing values or values outside the scale range
(`geom_ribbon()`).
```

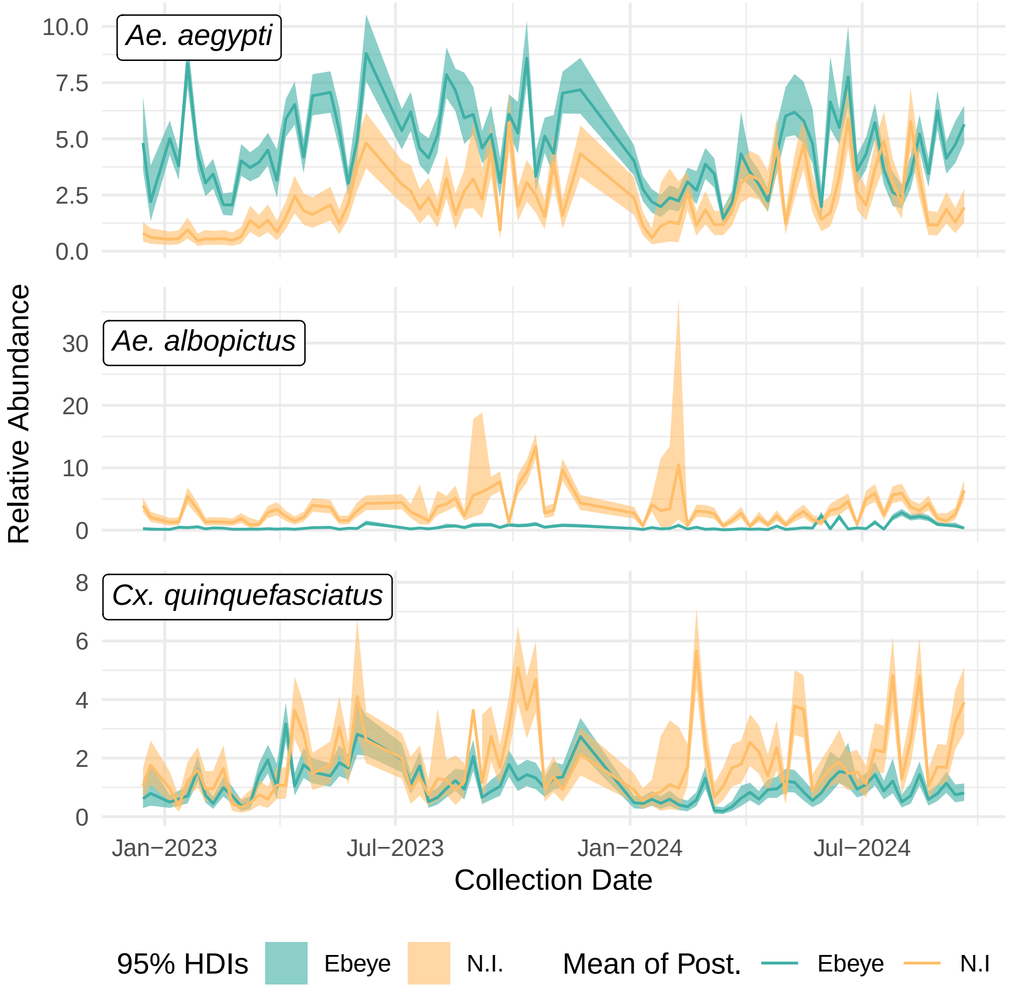

Figure S1: Abundance dynamics in Ebeye and the North Islets.

Regresssion tables

In Table S1, Table S2, and Table S3, we present the regression tables for the corresponding species. For further detail see article.

Table S1: *Ae. aegypti* Regression Table

Parameter	Estimate (log scale)	exp (Estimate)	95% HDI exp(Estimate)
Intercept	-0.93	0.42	0.17–0.75
β_1(log(Rain))	0.38	1.30	1.18–1.42
β_2(Wind)	0.00	1.00	0.95–1.06
β_3(Ebeye)	1.08	3.04	1.63–4.56
σ_{site}	0.70	2.02	1.72–2.37
σ_{week}	0.33	1.39	1.29–1.50
σ	0.70	2.02	1.72–2.37

Note:
Estimate = most probable value (mean of the posterior); exp(Estimate) = $e^{\beta_{2,3}}$ excepting rain. Rain, in power law scale, = 2^{β_1}; 95% HDI exp(Estimate) = 95% High Density Intervals of the transformed MCMC samples.

Table S2: *Ae. albopictus* regression Table

Parameter	Estimate (log scale)	exp (Estimate)	95% HDI exp(Estimate)
Intercept	0.02	1.25	0.19–2.89
β_1(log(Rain))	0.40	1.33	1.09–1.57
β_2(Wind)	-0.10	0.91	0.81–1.00
β_3(Ebeye)	-2.49	0.09	0.04–0.14
σ_{site}	0.92	2.53	2.01–3.13
σ_{week}	0.70	2.03	1.77–2.34
σ	0.92	2.53	2.01–3.13

Note:
Estimate = most probable value (mean of the posterior); exp(Estimate) = $e^{\beta_{2,3}}$ excepting rain. Rain, in power law scale, = 2^{β_1}; 95% HDI exp(Estimate) = 95% High Density Intervals of the transformed MCMC samples.

Table S3: *Cx. quinquefasciatus* regression Table

Parameter	Estimate (log scale)	exp (Estimate)	95% HDI exp(Estimate)
Intercept	-1.26	0.32	0.07–0.62
β_1(log(Rain))	0.43	1.35	1.19–1.52
β_2(Wind)	0.00	1.00	0.92–1.07
β_3(Ebeye)	-0.67	0.53	0.24–0.86
σ_{site}	0.83	2.31	1.85–2.81
σ_{week}	0.44	1.56	1.39–1.73
σ	0.83	2.31	1.85–2.81

Note:
Estimate = most probable value (mean of the posterior); exp(Estimate) = $e^{\beta_{2,3}}$ excepting rain. Rain, in power law scale, = 2^{β_1}; 95% HDI exp(Estimate) = 95% High Density Intervals of the transformed MCMC samples.

The Impact of rain, wind, and demographics on *Ae. aegypti*

As described in Table S1, rain was statistically different from one (2^{β_1}) = 1.30[1.18–1.42]) , indicating that rainfall explained some of the variation observed in the relative abundance. Rain was back-transformed using 2^{β} because the relationship to the expected mean is $E[Y] \propto rain^{\beta_1}$; 2^{β_1} is the multiplicative factor by which the expected count changes when rain doubles. All together, when rain doubles the mean abundance of *Ae. aegypti* is expected to increase by ~30%.

The Wind variable did not have a significant effect on the relative abundance because the uncertainty interval overlaps one ($\exp(\beta_2)$ = 1.00[0.95–1.06]). Finally, Ebeye (a binomial variable),had a significant effect on the abundance ($\exp(\beta_3)$ = 3.04[1.63–4.56]).

Based on the model results we can say that *Ae. aegypti* relative abundance equation was $log(\lambda)$ = -0.93 + 0.38 × log(Rain) + 1.08 × Ebeye site. The adjusted variables are plotted against the obseved catches in Figure S2.

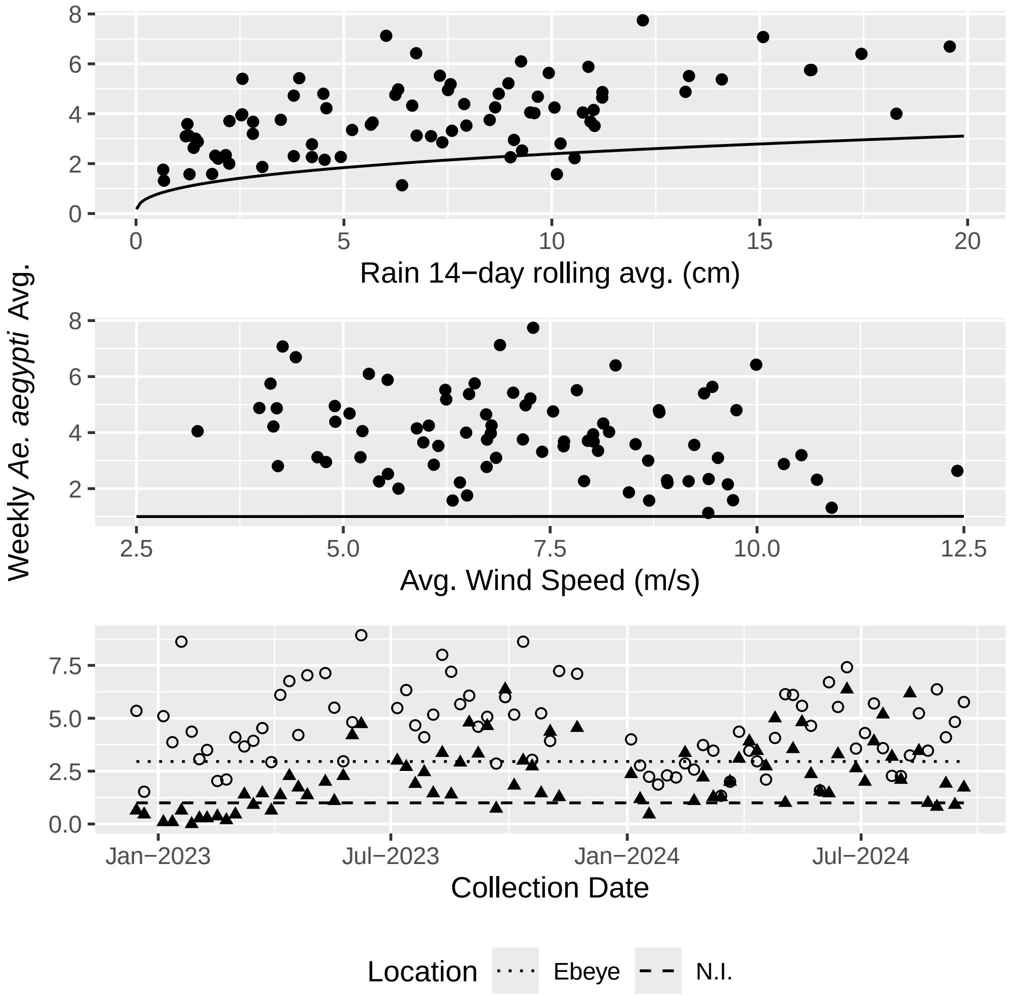

Figure S2: Modeled rain, wind, and location vs *Ae. aegypti* observed catches.
